# Next steps for transrectal ultrasound-guided prostate biopsy - where microvascular flow imaging plays a role

**DOI:** 10.1590/0100-3984.2025.58.e10

**Published:** 2025-12-22

**Authors:** Andrea Cavalanti Gomes, Rodrigo Salvador Vivas Cardoso

**Affiliations:** 1 Instituto de Radiologia do Hospital das Clínicas da Faculdade de Medicina da Universidade de São Paulo (InRad/HC-FMUSP), São Paulo, SP, Brazil

Prostate cancer is characterized by increased microvascularization and abnormal vascular architecture, which can be visualized using advanced ultrasound modalities. Microvascular imaging (MVI) and contrast-enhanced ultrasound (CEUS) have demonstrated improved sensitivity over conventional color Doppler ultrasound in detecting microvascular flow associated with malignancy. MVI-guided targeted biopsy has been shown to increase the detection rate of clinically significant prostate cancer (csPCa) in comparison with systematic biopsy alone^(1-3)^.

Multiparametric ultrasound approaches, which comprise B-mode ultrasound, elastography, MVI, and CEUS, further enhance the visualization of microvascular and tissue characteristics, improving the accuracy of targeted biopsies. Current evidence demonstrates that MVI-, CEUS-, and high-frequency micro-ultrasound (MicroUS)-guided biopsies all improve the detection rate of csPCa in comparison with conventional transrectal ultrasound (TRUS)-guided systematic biopsy^(1-7)^.

Conventional TRUS remains limited in its ability to distinguish between benign and malignant lesions solely on the basis of grayscale or standard Doppler imaging, because benign conditions such as prostatitis and benign prostatic hyperplasia can also increase vascularity^(8)^. Therefore, integrating advanced microvascular imaging techniques into the TRUS-guided biopsy workflow represents a promising strategy to improve diagnostic yield and risk stratification in prostate cancer^(1-8)^. These advanced techniques are increasingly recognized as promising alternatives or complements to magnetic resonance imaging (MRI), especially in settings in which MRI is limited or contraindicated^(3,5,9)^.

It is satisfying to witness increasing evidence, as shown by the article “Assessment of microvascular flow by Doppler in prostate biopsy: correlation with the Gleason score”^(1)^, published in Radiologia Brasileira, that MVI provides superior visualization of microvascularity and is associated with higher Gleason scores at the site of interest, and that MVI-guided targeted biopsy yields a significantly higher detection rate for csPCa than does systematic TRUS-guided biopsy. These advantages must be taken into account when prostate biopsy is performed, especially in challenging cases after multiparametric MRI. The conclusion of the article highlights the importance of making these technologies more accessible, aiming for the widespread use of these ancillary tools (as commented upon above) in the context of TRUS-guided biopsy, resulting in a diagnosis that is more efficient and patient care that is more assertive.

Future directions include the use of CEUS and MicroUS as ancillary methods, adding value to elastography and MVI. The use of CEUS enhances the sensitivity and overall accuracy of prostate cancer detection, particularly in patients with prostate-specific antigen levels in the diagnostic gray zone (4-10 ng/mL), and increases the rate of positivity per core biopsy and overall detection of csPCa^(3,5,10)^. Meta-analyses have confirmed that CEUS-guided targeted biopsy outperforms conventional TRUS-guided systematic biopsy in both sensitivity and overall accuracy for csPCa detection^(3)^. Another upcoming tool to integrate the multiparametric study and biopsy of the prostate is MicroUS, which, by utilizing a 29-MHz transducer, achieves real-time, high-resolution imaging and enables targeted biopsy of suspicious lesions. Systematic reviews and meta-analyses have shown that MicroUS-guided biopsy detects more csPCa and fewer clinically insignificant cancers than does conventional systematic biopsy, thus reducing overdiagnosis^(5,7,9,10)^. In addition, MicroUS identifies MRI-invisible lesions, further increasing the diagnostic yield^(10)^.

In summary, MVI-, CEUS-, and MicroUS-guided biopsies all demonstrate superior or at least noninferior diagnostic accuracy for clinically significant prostate cancer in comparison with conventional TRUS-guided biopsy, with improved sensitivity and specificity, as well as reduced detection of indolent disease^(1-3,6,9,10)^.

There is a need for further studies to confirm the importance of introducing these new technologies that evaluate the microvascularization of prostatic lesions during biopsy and allow the identification of clinically significant neoplasms (Gleason score ≥7), so that they may become more widely disseminated and accessible, especially in challenging cases and to guide cognitive fusion biopsies after multiparametric MRI.
